# Numerical Investigations of Hepatic Spheroids Metabolic Reactions in a Perfusion Bioreactor

**DOI:** 10.3389/fbioe.2019.00221

**Published:** 2019-09-12

**Authors:** Fatemeh Sharifi, Bahar Firoozabadi, Keikhosrow Firoozbakhsh

**Affiliations:** School of Mechanical Engineering, Sharif University of Technology, Tehran, Iran

**Keywords:** hepatocytes, spheroids, bioreactor, oxygen concentration, modeling, metabolic functions

## Abstract

Miniaturized culture systems of hepatic cells are emerging as a strong tool facilitating studies related to liver diseases and drug discovery. However, the experimental optimization of various parameters involved in the operation of these systems is time-consuming and expensive. Hence, developing numerical tools predicting the function of such systems can significantly reduce the associated cost. In this paper, a perfusion-based three dimensional (3D) bioreactor comprising encapsulated human liver hepatocellular carcinoma (HepG2) spheroids are analyzed. The flow and mass transfer equations for oxygen as well as different metabolites such as albumin, glucose, glutamine, ammonia, and urea were solved in three different domains, i.e., free flow, hydrogel, and spheroid porous media sections. Since the spheroids were encapsulated inside the hydrogel, shear stress imposed on them were found to be less than tolerable thresholds. The predicted cumulative albumin concentration over the 7 days of culture period showed a good agreement with the experimental data. Based on the critical role of oxygen supply to the hepatocytes, a parametric study was performed and the effect of various parameters was investigated. Results illustrated that convection mechanism was the dominant transport mechanism in the main-stream section contrary to the intra spheroids parts where the diffusion was the prevailing transport mechanism. In the hydrogel parts, the rate of diffusion and convection mechanisms were almost identical. As expected, higher perfusion rate would provide high oxygen level for the cells and, smaller spheroids with a diameter of 100 μm were at the low risk of hypoxic conditions due to short diffusive oxygen penetration depth. Numerical results evidenced that spheroids with diameter size >200 μm at low porosities (ε = 0.2–0.3) were at risk of oxygen depletion, especially at locations near the core center. Therefore, these results could be beneficial in preventing hypoxic conditions during *in vitro* experiments. The presented numerical model provides a numerical platform which can help researchers to design and optimize complex bioreactors and obtain numerical indexes of the main metabolites in a very short time prior to any fabrications. Such numerical indexes can be helpful in certifying the outcomes of forensic investigations.

## Introduction

Drug development researches require strict toxicology that postulates the implication of numerous *in vivo* and *in vitro* studies (Esch et al., 2011). However, manufacturing effective drugs is often hampered by weak predictive preclinical experiments to accurately and reliably model the effects of drug compounds inside the human body, leading to low efficacy of drug development processes (Esch et al., 2011; Huh et al., 2012). Due to the role of the liver in the metabolism of drugs and exogenous chemical compounds, miniaturized human cell-based bioreactors mimicking the environment of the human's liver has emerged as a promising alternative tool for complementing the existing drug discovery approaches (Gebhardt, 1992; Groneberg et al., 2002; Baudoin et al., 2011). Apart from vital detoxification effects, the liver has other indispensable functions such as glucogenesis, lipid metabolism, and protein metabolism. Blood plasma protein, particularly albumin, is synthesized mostly by the liver (Gebhardt, 1992; Baudoin et al., 2011). This protein is essential in oncotic pressure maintenance and also participate as a transporter for fatty acids and steroid hormones (Baudoin et al., 2011). Furthermore, many substantial catabolites removal happens in the liver. For instance, highly toxic ammonia breakdowns to urea primarily occur in the liver, which is then excreted as urine by kidneys (Gebhardt, 1992). Majority of the aforementioned functions are carried out by the main parenchymal cell type of the liver, i.e., hepatocytes (Barrett et al., 2006). Metabolism for the vast majority of drugs and chemicals happens in the liver in which toxic metabolites are converted into relatively less harmful compounds for eventual urinary excretion (Barrett et al., 2006). It is noteworthy that acute drug-induced liver failure is one of the major reasons for post-market drug removal. Consequently, persistent exposure of hepatocytes to exogenous compounds makes them optimal candidates for toxicology and drug screening studies.

Thus, engineering liver-on-a-chip platforms for the purpose of drug discovery and toxicity analysis has been the subject of various investigations (Khetani and Bhatia, 2008; Baudoin et al., 2011). On the contrary to current *in vitro* models utilizing two dimensional (2D) cellular cultures, liver on-chip platforms typically accommodate 3D cell cultures (organoids), are perusable and can utilize human cells (Maguire et al., 2009; Ramaiahgari et al., 2014; Ahmed et al., 2017). Liver slices or biopsies may be the first 3D versatile model which intimately resemble the complex *in vivo* cytostructure but they rapidly lose their viabilities for about 1 day (van Midwoud et al., 2010; Soldatow et al., 2013). Furthermore, the rate of drug metabolism in liver slices are lower in comparison to the isolated hepatocytes (Ekins et al., 1995; Yu et al., 2001). Therefore, liver slices may not be a suitable alternative to be used for a high-throughput drug screening. Isolated hepatocytes entrapped in collagen sandwiches (Dunn et al., 1991; LeCluyse et al., 1994; Kern et al., 1997), hollow fiber scaffolds (Bao et al., 2011; Skardal et al., 2012; Godoy et al., 2013), hydrogels (Hou et al., 2010, 2011b; Kim et al., 2011) and hepatospheres are well-known 3D models of improving hepatocyte viability and increasing the cell polarization toward creating a more complex network leading to more liver-like models. The use of hepatic spheroids has been shown to maintain liver viability and liver-specific functions such as albumin production, urea synthesis for longer time intervals respect to the other aforementioned cultures (Ramaiahgari et al., 2014). In spheroids, cells are closely in contact with each other and produce their own extracellular matrix (ECM), which result in retaining most of their cell-cell and cell-ECM contacts in comparison to the majority of the three-dimensional culture methods (Godoy et al., 2013).

Despite the efforts in making liver-on-a-chip platforms, there are still no guidelines for determining the proper design parameters, including perfusion rate, number of cells, size of the spheroids, etc. One of the critical challenges of 3D hepatic cultures is supplying adequate oxygen and nutrition to the cell surface (Godoy et al., 2013; Hsu et al., 2014). The rate of oxygen consumption of hepatocyte in comparison to the other cell types is high due to the high metabolic activity of these cells (Ramaiahgari et al., 2014). Also, oxygen solubility in culture media at 37°C is low (Yu et al., 2001). Supplying sufficient oxygen to the hepatic spheroids becomes more severe because the abovementioned oxygen transport obstacles are accompanied by diffusion dominant transport mechanism inside the spheroid, which becomes particularly serious in spheroids with a larger diameter. Consequently, determining the oxygen distribution within liver-on-a-chip models comprising cells in the form of hepatic spheroids has remained an important factor, which is extremely hard to optimize experimentally.

Dynamic systems such as the perfusion bioreactors can be a great help since perfusion continuously supplies oxygen and nutrition to the cells and simultaneously removes metabolic waste, leading to improve viability and life span of the hepatocytes. However, perfusion of media inside the bioreactor will impose undesirable flow-induced shear stress on the cells, which can be detrimental or even cause cell detachment (Hou et al., 2011a). Therefore, there must be a balance between the culture medium flow rate and the oxygen supply for the cells (Hsu et al., 2011; Dumont et al., 2018).

Till now, several mathematical methods have been developed to investigate the effect of shear stress and mass transfer at the cellular surface (Curcio et al., 2007; Hsu et al., 2014; Bhise et al., 2016; Khakpour et al., 2017). Transport of oxygen inside the spheroids with different sizes cultured in a rotating wall system under both gas permeable and gas impermeable conditions was investigated by Curcio et al. (2007). Their results showed that spheroids with sizes larger than 200 μm were suffered from oxygen depletion. Khakpour et al. (2017) investigated the oxygen transfer to hepatospheres inside the hollow fiber membrane bioreactor, and the effect of various parameters such as perfusion rate, porosities, spheroid sizes, etc. were investigated. Optimal flow condition and oxygen concentration gradient were calculated numerically in a bioreactor containing hepatic spheroids encapsulated in hydrogel under continuous flow by Bhise et al. (2016). Spheroids in their bioreactor showed considerable viability and functionality over 30 days of culture periods (Bhise et al., 2016). Their results obtained from analyzing and monitoring the concentration of the secreted biomarkers such as albumin, alpha-1 antitrypsin (A1AT) indicated that the hepatic spheroids in their bioreactor remained functional over 30 days of culture periods (Bhise et al., 2016). In another study, Hsu et al. developed a mathematical model of a bioreactor in which a monolayer of HepG2 cells was cultured at the bottom (Hsu et al., 2014). Hydrodynamic equations, along with mass transfer equation were solved to obtain flow characteristics and metabolites concentration, respectively. Despite abovementioned efforts to model the shear stress and oxygen levels at the cellular surface, there is still lack of enough literature to mathematically incorporating intracellular metabolic activities of hepatocytes to their related metabolites in the extracellular environment via governing mass transfer in a bioreactor to quantitatively investigate the effect of different parameters. Such models provide an analytical platform for design, optimization of the bioreactor, and help researchers in perceiving better the interplay between bioreactor design parameters and liver-specific functions. This mathematical system also provides rapid analyses which can be helpful in the disciplines like forensic bioengineering where immediate and accurate results of trace sampling are crucial. Results from the presented numerical platform can also be used in better interpreting and certifying the outcomes of investigations. Various drug pathways like depressants, stimulants, analgesics, and psychotomimetics can be added to this numerical liver-on-a-chip; and hence, providing the results which can be used as a guide for testifying the related findings of drug abuse and forensic toxicology investigations. Also, the numerical system can be useful in prediction and estimation on the concentration of the species wherein some cases, only small amount of samples might be gathered in the victim scene. High-speed result of different concentrations of the substance can be provided using this numerical liver-on-a-chip platform.

**Figure 1:**
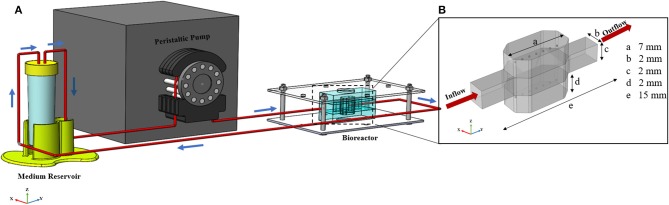
General configuration of liver spheroid-on-a-chip model. **(A)** Schematic of the bioreactor. Bioreactor composed of two spheroid chambers and a media channel. Spheroids were placed at the certain position inside the hydrogel. Flow direction is depicted with blue arrows. Flow enters from the middle channel and permeates through both upper and lower hydrogel chambers. The bioreactor was connected to the reservoir, and the whole system was run using a peristaltic pump. **(B)** Dimensions of the different section of bioreactor. The dimension of the upper and lower sections is similar.

In this study, we developed a numerical model based on the 3D culture of hepatic encapsulated spheroids in miniaturized chambers under continuous perfusion (Figure 1A). Optimized working conditions of the bioreactor were obtained in terms of sufficient oxygen delivery to the cellular surface and protecting the cells from high levels of shear stress. In the first step, fluid flow distribution inside the bioreactor is calculated. Transport phenomena are considered in different sections of the bioreactor, i.e., free flow, porous flow, spheroids, and single cell-level, providing a powerful tool mimicking a liver-on-a-chip system prior to any fabrications. Through the present computational multiscale model, researchers can test multiple designs and observe the effect of various parameters with real-time monitoring of the pivotal phenomena of dynamic nature of model under investigation in cost and time-effective manner. Additionally, mass transfer equations are incorporated into hydrodynamics equations to measure the concentration of different metabolites. Oxygen concentration and other main metabolite functions of the hepatocytes such as albumin production, glucose, and glutamine consumptions, ammonia and urea generation are obtained in subcellular level inside the implicitly modeled hepatocytes aggregated as a spheroid configuration using a mathematical model. Since sufficient oxygen availability is a necessary condition for the cellular viability, metabolites concentrations were calculated by incorporating the effect of oxygen tension on metabolite concentration. The above metabolites are diffused into or out of the spheroids through the hydrogel. The concentration distribution of each metabolite obtained in each spheroid and the whole domain. The model shows a great promise as the simulation results calculated for albumin over 4 weeks of culture showed good compatibility with experimental data of liver albumin production level. Moreover, in the final section, a parametric study has been carried out and the effect of different prevailing parameters on oxygen transfer to hepatocyte spheroids are investigated. Oxygen concentration is chosen as the main optimization criterion among other investigated metabolites for the parametric study because hepatocytes are highly susceptible to the oxygen level and they drastically lose their liver-specific functionalities under the effect of inadequate oxygen concentration.

## Methodology

The methodology used for modeling distribution of mass, flow, and concentration is illustrated in the following sections. Since the problem under investigation is composed of multi-physics, governing equations of the fluid flow and concentrations are coupled. The solution of continuity, hydrodynamic, and mass transfer equations are obtained using 3D finite element method. The Galerkin method was used in order to approximate the non-linear partial differential governing equations with a system of ordinary differential equations. The equivalent weak formulations of the Navier–Stokes, and convection-diffusion equations were achieved by applying the boundary conditions, followed by discretization of the weak forms in a finite-dimensional subspace. An Eulerian approach was chosen for the one-dimensional, time-dependent domain.

The geometry of the device is composed of three different chambers; two side chambers containing hydrogel in which hepatic spheroids are encapsulated and one middle chamber through which medium and nutrition flow. Schematic view of the bioreactor, including dimensions of different parts is depicted in Figure 1B. The overall bioreactor was connected to the reservoir, and the whole system was run using a peristaltic pump (Figure 1A).

**Figure 2:**
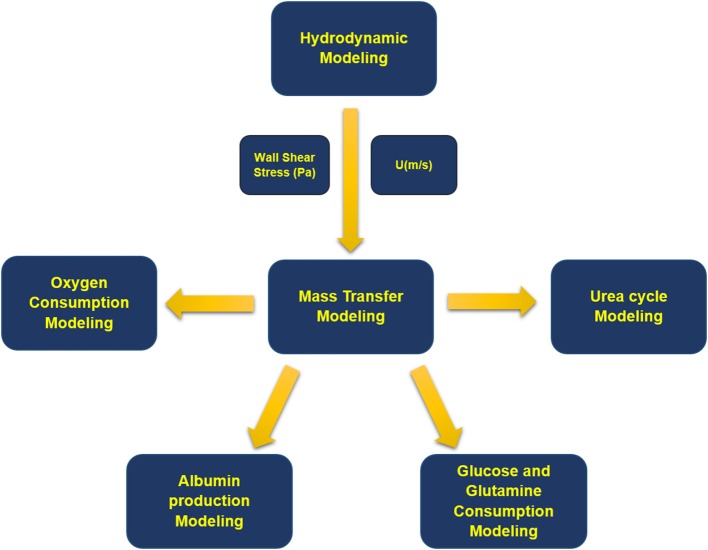
Block diagram of the modeling process. First, fluid flow equations were solved, and velocity distribution and wall shear stress inside the bioreactor were obtained. Next, mass transfer equations were solved for oxygen, albumin, glucose, glutamine, ammonia, urea, and their related metabolites.

Modeling methodology of the presented simulation is shown in a schematic configuration in Figure 2 denoting the coupling between governing equations describing the multiphysical nature of the problem. Fluid flow dynamics is coupled with mass transport and reactions inside the bioreactor. First, equations of continuity and momentum are solved, and fluid velocity distribution inside each section of the bioreactor is obtained. Then, for each concentration, mass transfer equation is computed along with related metabolic reactions.

### Fluid-Dynamics Model

**Figure 3:**
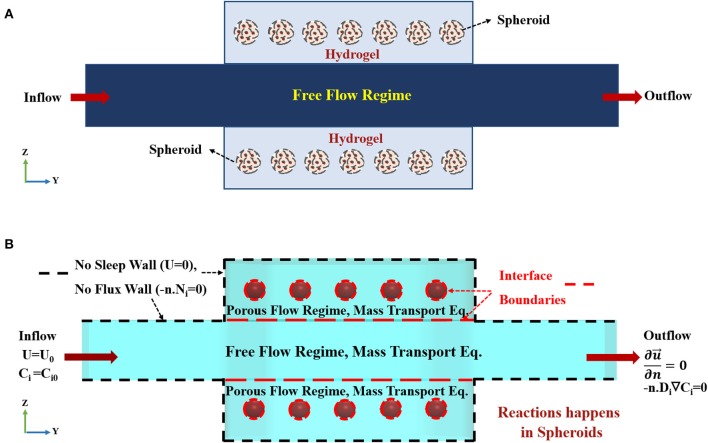
Concept schematic of the modeled geometry. **(A)** Schematic front view (Z-Y) of the bioreactor. The bioreactor is composed of two hydrogel chambers containing spheroids and a central main chamber. Medium flows into the main-stream and then spreads to the hydrogel compartments. **(B)** The imposed boundary conditions used for current simulation. Constant inlet velocity and constant initial concentration for species are set as the inlet boundary conditions for hydrodynamics and mass transfer equations, respectively. No slip (zero velocity) and no flux boundary conditions are applied on the boundaries marked by black dashed lines, since there is no penetration at the solid side walls of the bioreactor. The dashed red lines show the interface boundaries between free and porous flow domains and hydrogel-porous spheroid domain where species and stress continuity boundary conditions are applied. Outflow boundary condition is set at the outlet.

Flow containing oxygen and nutrition enters via the inlet port of the bioreactor at a prescribed volumetric flow rate. The top and the bottom surface of the fluidic channel, depicted in Figure 3A, are in contact with hydrogel. The hydrogel in this model is considered as a uniform, isotropic porous medium. Oxygen and nutrition will permeate via constant perfusion throughout the hydrogel due to its porous characteristics. Thereupon, the governing equation of fluid flow, mass transfer, and cell kinetics are coupled. The hydrodynamic modeling of the bioreactor is divided into two parts, i.e., free flow and porous flow regimes, based on their governing equations (Figure 3A and Figure S1). Inlet volumetric flow rate is 1 mlit/min, declaring that Reynolds number (Re) <2000, and the fluid flow regime is laminar inside the channel (For further explanation, please check Supplementary Materials). Based on the assumptions mentioned above, the governing equation demonstrating the flow of fluid and two hydrogel chambers will be Navier-Stokes and Stokes-Brinkman equations, respectively (Truskey et al., 2004; Figure S1A). Equations of continuity, momentum for free and porous flow regimes can be written as:

(1) $$\begin{array}{rc} \nabla \cdot \vec{\text{u}} & =0 \end{array}$$

(2) $$\begin{array}{rc} \rho \left(\vec{\text{u}}\cdot \nabla \vec{\text{u}}\right) & =\nabla \cdot \left[-\text{pI}+\mu \left(\nabla \vec{\text{u}}+{\left(\nabla \vec{\text{u}}\right)}^{\text{T}}\right)\right] \end{array}$$

(3) $$\begin{array}{l} \frac{\mu }{K}\vec{u}=\frac{1}{\rho }\nabla \cdot [-pI+\frac{\mu }{\varepsilon }\left(\nabla \vec{u}+{\left(\nabla \vec{u}\right)}^{T}\right)\\ \;-\left(\nabla \cdot \vec{u}\right)I] \end{array}$$

where ρ, μ, and *p* are fluid density, viscosity and pressure, respectively. *K* is the permeability, and ε is porosity in the porous flow regime, i.e., representing hydrogel and spheroids sections. $\vec{u}$ denotes three-dimensional fluid velocity vector $\vec{u}=$*(u, v, w)*. It is assumed that the solution is an incompressible, isothermal, Newtonian fluid. Since all of the nutrition inside the cell culture medium is dissolved in aqueous-based liquid, all of the fluid properties are assumed to be similar to the properties of water at the same temperature. The schematic of the boundary conditions used in the present simulation is depicted in Figure 3B. The above equations are coupled via two stress boundary conditions at the interface of the fluidic and two hydrogel chambers (For further explanation, please check Supplementary Materials). Here, in this model, hepatic spheroids with different diameters of 100 to 400 μm are located at the specific places inside the hydrogels. Spheroids are modeled again as a uniform, homogeneous porous medium. Boundary condition on each wall of the bioreactor is assumed to be zero velocity, representing no-slip boundary condition (Figure 3B). Since each spheroid is modeled as a porous sphere, hence, the velocity distribution inside the spheroid is obtained again by solving Stokes-Brinkman equation similar to the hydrogel compartments. Porosity and permeability of the spheroids are calculated based on their cellular density, which will be described further in details. Constant inlet flow velocity $(\vec{u}=u_{in})\;$at the inlet and outflow boundary condition $\left(\partial \vec{u}/\partial n=0\right)$, where **n** is a normal vector, at the outlet of the fluid flow channel are assumed for the hydrodynamic boundary conditions. The boundary condition at the outlet implicates that flow is fully developed (Figure 3B).

The shear stress on the cell surface can be measured by

(4) $$\begin{array}{r} \tau_{s}=\mu \frac{\partial \vec{\text{u}}}{\partial \text{n}} \end{array}$$

**n** is a normal vector of the cell surface. Parameters used for the simulation of fluid flow are given in Table 1.

**Table 1:** Material properties and constants used in hydrodynamic equations.

**Simulation parameter-symbol [SI unit]**	**Value**	**Source**
Density of the medium-ρ [kg/m^3^]	1000	Munson et al., 2014
Viscosity of the fluid-μ [Pa-s]	1.002e-3	Munson et al., 2014
Fluid velocity at inlet-u_in_ [mm/s]	4	Computed
Porosity of Hydrogel-ε_hyd_	0.7	Mai et al., 2014; Yue et al., 2015
Porosity of spheroid-ε_sph_	0.2	Ahmed et al., 2017
Water self-diffusivity-D [m^2^/s]	2.27 × 10^−9^	Merdaw et al., 2010
Molecular weight of water-M [gr/mol]	18	Hsu et al., 2014
Universal gas constant- R_g_ [J/(mol-K)]	8.314	Merdaw et al., 2010
Mean pore diameter of the hydrogel-d-_hyd_ [μm]	50	Bodenberger et al., 2016

### Mass Transfer Modeling

In this study, the distribution of each metabolite's concentration inside the spheroids, hydrogel and in the main section are obtained using mass transfer equation, i.e.,

(5) $$\begin{array}{r} \frac{\partial {\text{C}}_{i}}{\partial \text{t}}+\vec{\text{u}}\cdot \nabla {\text{C}}_{\text{i}}=\nabla \cdot \left({{\text{D}}_{\text{i}}\text{C}}_{\text{i}}\right) \end{array}$$

for the hydrogel compartments and the main section and

(6) $$\begin{array}{r} \frac{\partial {\text{C}}_{\text{i}}}{\partial \text{t}}+\vec{\text{u}}\cdot \nabla {\text{C}}_{\text{i}}=\nabla \cdot \left({{\text{D}}_{\text{i}}\text{C}}_{\text{i}}\right)+{\text{R}}_{\text{i}} \end{array}$$

for the spheroid parts, where *C*_*i*_ is the concentration of species, *D*_*i*_ represents species diffusion coefficient inside substrate solution, $\vec{\text{u}}$ is the velocity vector obtained from the hydrodynamic equations, *i* corresponds to the specific species and, *R*_*i*_ indicates the reaction rates of each species which specifies respected metabolic reactions of the cells (Figure S1B). The diffusion coefficient of metabolites inside the hydrogel is correlated to its counterparts in the medium substrate by an equation, which will be defined later. It is shown that the metabolite diffusion coefficient inside the hydrogel is increasing directly with porosity and has an inverse relation with the tortuosity of the hydrogel (Hou et al., 2010). The species modeled in this study are oxygen, albumin, glucose, glutamine, ammonia, and urea. At the inlet, constant inlet concentration ***C***_***i***_**=*C***_***i*.*in***_ and outflow boundary condition at the outlet;

(7) $$\begin{array}{r} -n\cdot {{\text{D}}_{\text{i}}\text{C}}_{\text{i}}=0 \end{array}$$

respect to each metabolite is considered as boundary conditions for mass transfer equation. Respected values for each metabolite is given in each section. No flux boundary condition is assumed for all walls, as well (Figure S1A). Parameters used for the simulation of mass transfer are given in Table 2.

**Table 2:** Parameters constants used in the mass transfer modeling.

**Simulation parameter-symbol [SI unit]**	**Value**	**Source**
Oxygen inlet concentration-C_O2, in_ [mol/m^3^]	0.28	Mazzei et al., 2010; Tourlomousis and Chang, 2016b; Fournier, 2017
Oxygen diffusivity in cell culture medium –D_O2_ [m^2^/s]	3.4 × 10^−9^	Khakpour et al., 2017
Number of HepG2 inside each spheroid-N_cell_	30 × 10^3^	Ahmed et al., 2017
Half saturation constant of HepG2 –K_m_ [mol/m^3^]	0.154	Ahmed et al., 2017
Maximum HepG2 oxygen uptake rate –V_m_ [mol/(s-cell)]	0.68 × 10^−15^	Ahmed et al., 2017
Henry's Constant for Oxygen-k_H_ [atm/(mol/L)]	932.4	Mazzei et al., 2010
Albumin inlet concentration-C_Alb, in_ [mol/m^3^]	0	Hsu et al., 2014
Glucose inlet concentration-C_Glu, in_ [mol/m^3^]	5.5	Hsu et al., 2014
Glutamine inlet concentration-C_Glut, in_ [mol/m^3^]	2	Hsu et al., 2014
Ammonia inlet concentration-C_NH3, in_ [mol/m^3^]	2	Laemmle et al., 2016
Albumin diffusion coefficient in the medium –D_Alb_ [m^2^/s]	6.1 × 10^−11^	Vogel, 1988
Glucose diffusion coefficient in the water –D_Glu_ [m^2^/s]	6.0 × 10^−10^	Stein and Litman, 2014
Glutamine diffusion coefficient in the medium –D_Glut_ [m^2^/s]	7.5 × 10^−10^	Longsworth, 1953
Ammonia diffusion coefficient in the water –D_NH3_ [m^2^/s]	1.86 × 10^−9^	Picioreanu et al., 1997

The hepatic cell density of the spheroid is a function of its porosity where a high packed spheroid containing a large number of hepatic cells will have the smaller void fraction or lower porosity; hence

(8) $$\begin{array}{r} \rho_{\text{cell}-\text{spheroid}}=\frac{1-\varepsilon_{\text{sph}}}{{\text{V}}_{\text{cell}}} \end{array}$$

ε_*sph*_ is the spheroid porosity, and V_cell_ is the volume of the cells (Khakpour et al., 2017). Furthermore, the effective diffusion coeffcient for each metabolite inside the porous media, *D*_eff_, is corresponded to its respected diffusion coefficient in the liquid phase,

(9) $$\begin{array}{r} {\text{D}}_{\text{eff}.\;\text{i}}=\frac{\varepsilon }{\tau }{\text{D}}_{\text{i}} \end{array}$$

which can be written for hydrogel sections and spheroids. τ and ε are represented tortuosity and porosity, respectively. Here Bruggeman (Vafai, 2015; Equation 10) with ε_*sph*_> 0.2 and Wakao-Smith (Merdaw et al., 2010; Equation 11) relations are used for calculating the tortuosity of the hepatic spheroid and hydrogel parts, respectively, i.e.:

(10) $$\begin{array}{r} \tau_{\text{sph}}=\frac{1}{\varepsilon_{\text{sph}}^{0.5}} \end{array}$$

(11) $$\begin{array}{r} \tau_{\text{hyd}}=\frac{1}{\varepsilon_{\text{hyd}}} \end{array}$$

The permeability of media in the hepatic spheroids is modeled using Carman Kozeny model (Shipley and Waters, 2011), in which hepatocytes are considered as a sphere with the diameter of *d*_*hep*_ = 16 μm,

(12) $$\begin{array}{r} {\text{K}}_{\text{s}p\text{h}}=\frac{\varepsilon_{\text{s}p\text{h}}^{3}{\text{d}}_{\text{h}e\text{p}}^{2}}{180\times {\left(1-\varepsilon_{\text{s}p\text{h}}\right)}^{2}} \end{array}$$

Also, the hydrogel permeability is calculated via Merdaw approximation (Merdaw et al., 2010) in which permeability of water inside the hydrogel was modeled. For the presented study, the fluid properties of culture media considered to be similar to the fluid properties of the water at 37°C. Hence, the Merdaw approximation for the presented model (Merdaw et al., 2010) becomes;

(13) $$\begin{array}{r} {\text{K}}_{\text{hyd}}=\frac{\text{DM}\mu }{\rho {\text{R}}_{\text{g}}\text{T}}\varepsilon_{\text{hyd}}^{2}+\frac{{\text{d}}_{\text{hyd}}^{2}}{11.25}\frac{\varepsilon_{\text{hyd}}^{3}}{180\times {\left(1-\varepsilon_{\text{hyd}}\right)}^{2}} \end{array}$$

where D, μ, M, and ρ are the water self-diffusivity, viscosity, molecular weight, and density at 37°C, respectively. R_g_ is the universal gas constant, T is temperature, ε_hyd_ is hydrogel porosity, and d_hyd_ is mean pore diameter of the hydrogel. These parameters are given in Table 1.

#### Oxygen Concentration

Inlet oxygen concentration inside the medium is obtained via Henry's law, which is relating dissolved oxygen concentration inside the medium to the oxygen partial pressure at 37°C and atmospheric pressure condition (Barrett et al., 2006; Khetani and Bhatia, 2008):

(14) $$\begin{array}{r} p_{o_{2}}=k_{H}C_{o_{2}} \end{array}$$

where *p*_*O*2_ denotes partial pressure of the oxygen, *k*_*H*_ is Henry's constant and *C*_*O*2_ represents the concentration of the oxygen inside the solution (Maguire et al., 2009). This concentration is assumed to be constant at the inlet since the PDMS (Polydimethylsiloxane) is permeable to the oxygen and oxygen pressure will always equilibrate with oxygen existed in the surrounding environment, i.e., inside the incubator. It is possible to modify the inlet oxygen concentration by changing the oxygen supply level of the incubator. The effect of this parameter will be investigated in the parametric study section. Three layers of the bioreactor are fixed via two PMMA (Polymethylmethacrylate) sheets; therefore, there is no oxygen diffusion from the top and bottom surface of the bioreactor.

Based on the previous studies (Gebhardt, 1992), it is shown that the oxygen consumption rate of hepatocytes depends on the available oxygen concentration in the vicinity of the cell surface (Gebhardt, 1992). Oxygen consumption rate is governed by the first order Michaelis-Menten equation, which is the best-known model relating the rate of hepatic oxygen consumption to the oxygen concentration inside the medium (Gebhardt, 1992):

(15) $$\begin{array}{r} {\text{R}}_{{\text{o}}_{2}}={\text{N}}_{\text{cell}}\times \frac{{\text{V}}_{\text{m}}\times {\text{C}}_{{\text{o}}_{2}}}{{\text{K}}_{\text{m}}+{\text{C}}_{{\text{o}}_{2}}} \end{array}$$

where *N*_*cell*_ is cell number for each spheroid, *V*_*m*_ is maximum oxygen uptake rate per cell and K_m_ denotes Michaelis-Menten constant indicating oxygen partial pressure at the point where oxygen uptake rate reaches half of its maximum amount. Parameters used in this section are given in Table 2.

#### Albumin Concentration

It is mentioned that the albumin production of HepG2 cells is related to the local oxygen concentration at the cell surface and shear stress sensed by the cell due to fluid flow. Albumin production rate can be modeled via

(16) $$\begin{array}{r} {\text{R}}_{Alb}={\text{N}}_{cell}\times \left({\text{e}}_{1}\ln\left(\tau_{\text{s}}\right)+{\text{e}}_{2}+{\text{e}}_{3}{\text{C}}_{{\text{o}}_{2}}\right)\times \left({\text{C}}_{{\text{o}}_{2}}>0.03\right)\\ \;\times \left({\text{C}}_{Glu}>0\right)\times \left({\text{C}}_{Glut}>0\right) \end{array}$$

where *R*_*Alb*_ is albumin production rate of hepatic cells and τ_*s*_ is the shear stress felt by cells (Hsu et al., 2014). The last three terms in the above equation indicating that albumin production requires sufficient oxygen and nutrition concentrations. Therefore, in a situation where the oxygen concentration is below 0.03 mol/m^3^, or concentration of glucose or glutamine is zero, albumin production will be zero. Constants used in Equation (16) are given in Table 3.

**Table 3:** Constants used in mass transfer modeling of Albumin, Glucose, and Glutamine (Hsu et al., 2014).

**Symbol [SI unit]**	**Value**
e_1_ [mol/(s-cell-Pa)]	−5.9 × 10^−22^
e_2_ [mol/(s-cell)]	2.1 × 10^−22^
e_3_ [m^3^/(s-cell)]	8.2 × 10^−21^
e_4_ [m^3^/(s-cell)]	−4.8 × 10^−16^
e_5_ [mol/(s-cell)]	1.2 × 10^−16^
e_6_ [mol/(s-cell)]	1.4 × 10^−16^
e_7_ [mol/(s-cell)]	2.9 × 10^−16^

#### Glucose and Glutamine Consumption

HepG2 cells consume Glucose and Glutamine of the nutrition media. It is shown that (Barrett et al., 2006) Glucose consumption rate of the HepG2 cells is inversely related to the oxygen availability of the cells where at high oxygen concentration, Glucose consumption of the HepG2 is low while at lower oxygen tension, it is found that these cells will take up more Glucose (Barrett et al., 2006). Therefore, the Glucose consumption rate of the HepG2 can be written as

(17) $$\begin{array}{r} {\text{R}}_{\text{Glu}}={\text{N}}_{\text{cell}}\times \left({\text{e}}_{4}{\text{C}}_{{\text{o}}_{2}}+{\text{e}}_{5}\right)\times \left({\text{C}}_{\text{Glu}}>0\right) \end{array}$$

where *e*_4_ and *e*_5_ are given in Table 3.

**Figure 4:**
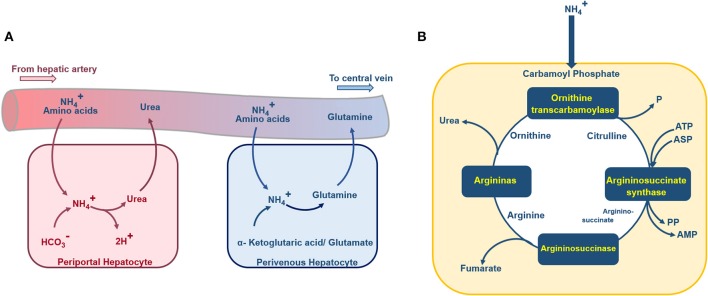
Schematic diagram of the urea cycle, ammonia detoxification, and glutamine synthesis inside a hepatocyte. **(A)** Periportal hepatocytes receive blood with high oxygen supply and nutrition from the portal vein and hepatic artery. In these hepatocytes, the ammonia detoxification is represented by the synthesis of the urea. Perivenous hepatocytes, aligned near the central vein, are supplied with less nutrition and low oxygenated blood, the ammonia detoxification here is accompanied by glutamine synthesis. **(B)** Ammonia enters the hepatocytes urea cycle as carbamoyl phosphate, passes through four enzymatic reactions and at final stage urea excretes to the blood.

Glutamine consumption rate (Figure 4A) of HepG2 is depended on the ammonia concentration inside the media. It is shown that (Baudoin et al., 2011) at the ammonia concentration of <10 mM, the glutamine consumption rate of the cells is 1.43 × 10^−16^ mols^−1^cell^−1^. At ammonia concentration of higher than 10 mM, the glutamine consumption rate will be 2.85 × 10^−16^ mols^−1^cell^−1^ (Baudoin et al., 2011). Hence, Glutamine consumption rate can be obtained via

(18) $$\begin{array}{r} {\text{R}}_{\text{Glut}}={\text{N}}_{\text{cell}}\times \left[\left({\text{e}}_{6}\times \left({\text{C}}_{{\text{NH}}_{3}}<10\right)\right)+\left({\text{e}}_{7}\times \left({\text{C}}_{{\text{NH}}_{3}}>10\right)\right)\right]\\ \;\times \left({\text{C}}_{\text{Glut}}>0\right) \end{array}$$

The above constants, *e*_6_, and *e*_7_, are given in Table 3.

#### Ammonia Production and Consumption

Ammonia and glutamate are generated by degradation of glutamine. Also, in the following steps, Glutamate dehydrogenase can lead to the formation of another molecule of ammonia from glutamate (Figure 4A; Baudoin et al., 2011). Therefore, during the degradation process of the glutamine, two molecules of ammonia will be generated. Based on the mentioned relation between glutamine degradation and ammonia formation, the rate of ammonia production is twice the rate of glutamine consumption (Hsu et al., 2014). Also, liver cells convert the available ammonia to urea through the urea cycle (Figure 4B). The detailed urea cycle reactions are given in Supplementary Materials. Hence,

(19) $$\begin{array}{r} {\text{R}}_{{\text{NH}}_{3}}={2\times \text{R}}_{\text{Glut}}\times \left({\text{C}}_{{\text{o}}_{2}}>0.03\right)-{\text{R}}_{{\text{NH}}_{3}.\text{c}} \end{array}$$

where the last term on the right-hand side of the equation denotes ammonia conversion to urea, which is modeled based on four main enzymatic reactions in the urea cycle (Kuchel et al., 1977). The detailed enzymatic reactions used for modeling urea generation are given in Supplementary Materials in detail.

## Results and Discussion

### Validation of the Numerical Method

**Figure 5:**
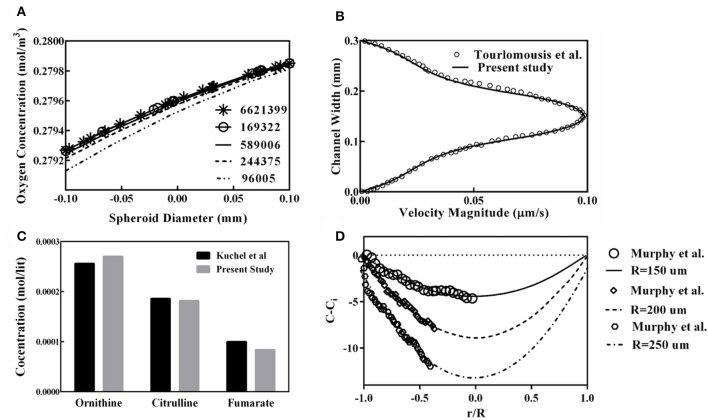
Validation and Grid study for evaluation of the presented code used in this study. **(A)** Oxygen concentration profile along the spheroid diameter for different mesh numbers. **(B)** Comparison of computed results of the fluid velocity inside the channel to those reported by Tourlomousis and Chang (2016b). **(C)** Comparison of the predicted metabolites participating in the urea cycle with the results given by Kuchel et al. (1977) **(D)** Oxygen concentrations are compared with the experimental data obtained by Murphy et al. (2017) for three different spheroid sizes.

The oxygen concentration profile along the spheroid diameter is plotted for different mesh numbers (Figure 5A), providing the mesh size independency of the obtained results of the simulation. From this figure, it can be inferred that results obtained from the grid with 589,006 elements are suitable for further studies and finer grid numbers do not show any significant discrepancies. Also, to validate the finite element code used for the present study, the generated code was used for replicating of the previous study remodeling carried out by Tourlomousis and Chang (2016b). Computed results of the fluid velocity inside the channel are compared with those reported by Tourlomousis and Chang (2016b) showing excellent compatibility (Figure 5B). Also, the results of the predicted metabolites participating in the urea cycle were modeled and compared with the results given by Kuchel et al. (1977). Three random metabolite concentrations are chosen and depicted in Figure 5C. Obtained results have <10% differences in comparison to the data given by Kuchel et al. (1977). For validating the oxygen concentration data predicted by the present simulation, calculated oxygen concentrations are compared with the reported experimental data for three different spheroid sizes, Figure 5D, representing a good agreement between computed oxygen concentration and experimental data given by Murphy et al. (2017).

### Hydrodynamic and Shear Stress

The main purpose of the perfusion system inside each microbioreactor is providing nutrition and oxygen to the cultured cells (Tanaka et al., 2006). Availability of a certain amount of oxygen is of great importance for cells to have normal metabolic activity. According to the literature (Evenou et al., 2010; Hsu et al., 2014), hepatocytes consume the highest amount of oxygen in comparison to the other cell types due to their high metabolic activities. Based on the low oxygen solubility and diffusivity in the culture medium, the concentration of oxygen is a critical factor in designing the bioreactors. Perfusion of the culture medium can enhance and control the oxygen supply to the cells by regulating the medium flow rate, which in turn imposes shear stresses on them. It is mentioned that shear stress affects metabolic functions and morphological configuration of the hepatocytes (Tanaka et al., 2006). Accordingly, there should be a balance between oxygen delivery to the cells and perfusion rate of the medium flow since high perfusion rate of culture media implies the excess amount of shear stress to the cells, which is also detrimental. Several studies indicated that hepatocytes viability under high shear stress conditions fell below that of hepatocytes cultured in static conditions (Park et al., 2008). It should be mentioned that the shear stress would affect hepatocyte functionality at values > 0.03 Pa (Tilles et al., 2001). Upon the hepatocyte's sensitivity to oxygen concentration values, optimization and parametric study carry out here for perfusion bioreactor has considered different aspects affecting oxygen supply to the hepatocytes. Perfusion rate is one of the efficacious parameters, where high flow rates have been proved to provide ample oxygen supply to the cells and on the other hand, imposes deleterious shear stress on the cell surface. Thereupon, the optimization in terms of the abovementioned parameters, which will be discussed further in the parametric study, signifies a delight balance between oxygen supply and shear stress.

**Figure 6:**
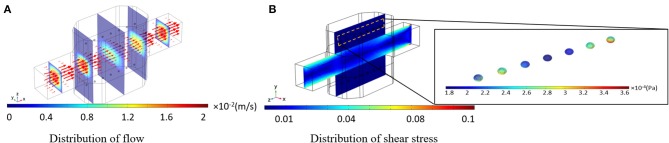
Results of the numerical simulation of fluid flow inside the bioreactor. **(A)** Fluid velocity distribution inside the bioreactor. Red vectors show the direction of the flow, which their length is correlated to the velocity magnitude **(B)** Shear stress distribution inside the whole bioreactor system. Shear stress imposed by the perfusion of the culture medium is below the threshold.

Culture medium velocity distribution is displayed in Figure 6A. The arrows are aligned from the inlet to the outlet of the bioreactor, tangential to the velocity of each point which length is corresponding to the velocity magnitude. It can be observed that the velocity vector at the centerline of the bioreactor is higher than the rest of the vectors, indicating a parabolic profile for the velocity inside the main channel of the bioreactor. Fluid flow velocity inside the hydrogel part of the bioreactor has small values, leading to low-value shear stresses applied on the spheroids hosted inside the hydrogel. Thus, the perfusion rate of culture medium for encapsulated cells can be higher than those cells directly exposed to the fluid flow domain. Shear stress distribution inside the bioreactor and shear stresses imposed on spheroids are shown in Figure 6B. The allowable shear stress tolerated by cells or required as a desirable factor inducing mechanotransduction effects is an intricate issue depending on many factors including cell type, cultural condition, culture period and elevated hepatic functions (Wang et al., 2010; Ebrahimkhani et al., 2014). The critical information from shear stress distribution (Figure 6B) is that shear stresses inside the hydrogels compartments and on spheroids are at values below the reported threshold of 0.03 Pa (Tilles et al., 2001). Such analysis is indispensable in perfusion bioreactors as cells are prone to a high level of shear stress.

### Oxygen Concentration

**Figure 7:**
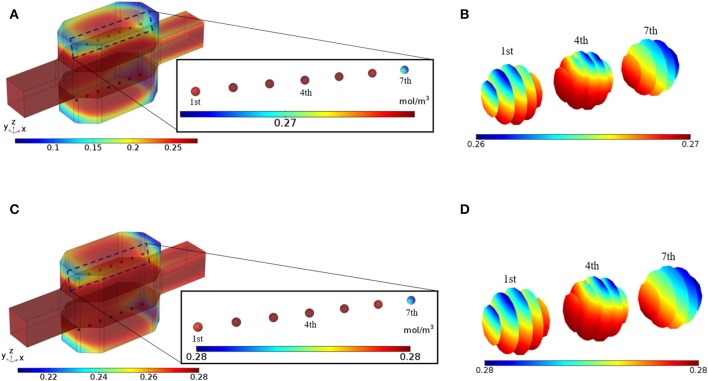
Results of the Oxygen concentration distribution inside the bioreactor. **(A)** The oxygen concentration in the bioreactor at t = 5 min. **(B)** Oxygen distribution inside the 1st, 4th, and 7th spheroids, t = 5 min **(C)** The oxygen concentration in the bioreactor at t = 10 min. **(D)** Oxygen distribution inside the 1st, 4th, and 7th spheroids t = 10 min.

The bioreactor is perfused with the medium under the *in vitro* standard conditions, i.e., 20% oxygen partial pressure at 37°C. Since spheroids are considered to be non-vascularized, it is possible to increase inlet oxygen concentration to prevent cells from oxygen deprivation. Such higher oxygen concentration is not detrimental to the hepatocytes since the oxygen concentration in the liver is between 4 and 9% (42–91 μmol/L) (Park et al., 2008). The effect of higher inlet oxygen concentration will be investigated in the parametric study section. Figure 7A shows oxygen concentration distribution inside the bioreactor after 5 min of perfusion, indicating that oxygen concentration in the vicinity of the hepatic spheroids is sufficient for the cells. Oxygen insufficiency at the center part of the spheroids is common since the transport mechanism for oxygen delivery to hepatocytes inside the spheroid is mainly based on diffusion. Due to the effect of the perfusion, oxygen supply at spheroid surfaces is improved, but it still is possible that in some part of the hepatic spheroids, oxygen level falls below the minimum oxygen supply necessary for the cells. In Figure 7B oxygen concentration distribution inside the first, fourth, and seventh spheroid at t = 5 min is presented, denoting that in the whole spheroids adequate oxygen supply is available for the hepatocytes. Oxygen is continuously consumed by the cells; therefore, oxygen tension might fall below the critical value [0.03 mol/m^3^ (Jungermann and Kietzmann, 2000)]. Oxygen distribution throughout the bioreactor and inside the hepatic spheroids at t = 10 min are displayed in Figures 7C,D, respectively which reflects that oxygen tension is also in an adequate level for the cells showing that perfusion can maintain sufficient oxygen supply to the hepatocytes resulting in preserving hepatocytes functionality. These results clarify that the hepatocytes aggregates inside the perfusion bioreactor are under the normal oxygenation. Greater oxygen tension can be supplied by applying higher oxygen level at the inlet or increasing the perfusion rate in this bioreactor since hydrogel in the cellular section of the bioreactor preserves the cells from a high level of shear stress exposure. A higher level of oxygen at the inlet can be obtained by increasing the oxygen supply inside the incubator. Furthermore, oxygen penetration from the PDMS sections of the bioreactor incessantly keeps oxygen tension at the maximum level, which also has a profound effect on sustaining hepatocyte-specific functions. The effect of this parameter will be analyzed in the parametric study.

### Albumin Concentrations

**Figure 8:**
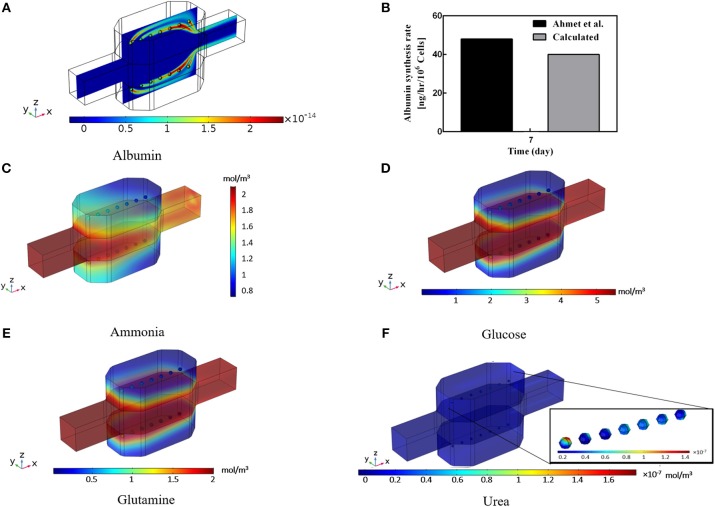
Results of the Albumin, Ammonia, Glucose, Glutamine, and Urea concentration inside the bioreactor. **(A)** Albumin concentration distribution inside the bioreactor at t = 10 min. **(B)** Cumulative albumin concentration at 7th day, showing a good agreement with the experimental data calculated by Ahmed et al. (2017). **(C)** Calculated ammonia concentration distribution. **(D)** Predicted glucose concentration distribution, **(E)** Glutamine concentration distribution. **(F)** Predicted urea production distribution at day 2 inside the bioreactor.

Albumin secretion analysis is generally one of the critical factors used by many studies in evaluating the functionality of the hepatocyte bioreactor (Glicklis et al., 2004; Anada et al., 2012; Bhise et al., 2016; Ahmed et al., 2017). It is proved that (Dullien, 2012) providing higher oxygen level to the hepatocytes will increase their albumin production rates whereas increasing shear stress levels imposed on cells by medium flow will decrease metabolic functionality and thereby the albumin production rate (Tan et al., 2013; Hsu et al., 2014). Hence, albumin production rate under the influence of two effective parameters, i.e., oxygen tension at the vicinity of the cells and shear stress sensed by them, is analyzed here via applying the method developed by Hsu et al. (2014). Results of the hepatocyte albumin production mapped on the central surface of the bioreactor at t = 10 min is displayed in Figure 8A. From this figure, it can be observed that albumin concentration is raised from zero to 2 × 10^−14^ mol/m^3^ in about 10 min of the culture duration. Also, the cumulative concentration of the albumin secretion at the outlet of the bioreactor over 7 days of the culture period is calculated (Figure 8B) and results are compared with experimental data of Ahmed et al. (2017), representing good agreement with the experimental data, though experimental results are slightly higher than the predicted value obtained by numerical simulation. This discrepancy might be based on the simplification made for generating the albumin production rate equation (Equaton 16), in which some other parameters affecting albumin synthesis in hepatocytes like nutritional and hormonal factors are ignored (Rothschild et al., 1977; Hutson et al., 1987).

### Glutamine, Glucose, Ammonia, and Urea Concentrations

Ammonia synthesis is one of the hepatocytes critical functions in which hepatocytes eliminate ammonia from the circulating system through the urea cycle leading to a non-toxic compound called urea. Figure 8C shows the predicted ammonia concentration distribution inside the incubator. Ammonia is removed via the urea cycle and generated in glutamine synthesis. Glucose is a necessary compound in culturing the hepatocytes. The distribution of glucose concentration in Figure 8D is also indicating the lower level of glucose respect to the inlet concentration. The negligible decrease of glucose concentration implies the low level of glucose consumption rate of the cells. This small low diminution of glucose is compensated by the relatively high concentration of the glucose at the inlet, providing sufficient glucose level for the cells. Distribution of the glutamine concentration is shown in Figure 8E, demonstrating the glutamine consumption at the spheroid surface. The glutamine level falls from its initial value due to the cellular intake. This small gradual decrease continues during the culture period and controls mainly by ammonia concentration inside the bioreactor (Equation 18). Urea production through urea synthesis is shown in Figure 8F denoting the small value of urea generated during 2 days of the culture period. Ammonia to urea conversion happens at the subcellular level, which is incorporated here for each hepatocyte and modeled through four enzymatic reactions (Supplementary Materials). Despite various assumptions for obtaining the aforesaid metabolites, predicted results provide precious insights into the different parameters for the researchers before performing any experiments.

Both calculated glucose and glutamine distributions are obtained based on the simplified methods developed by Hsu et al. (2014). More detailed metabolic relations should be included to have a more accurate and sensitive prediction, similar to the method used here for urea cycle prediction, so that their respected subcellular functions are incorporated. Therefore, glutamine and glucose distribution though providing a general reasonable overview and initial estimation, may not be considered as a sensitive tool in the evaluation and analysis of bioreactor design. On the contrary, results of oxygen, albumin, ammonia, and urea concentration are showed the promising indexes which can be utilized in assessing the efficiency and performance of the perfusion bioreactor. In this regard, based on the results of the oxygen distribution, a parametric study is conducted in the next section, and the effect of various design and working parameters are analyzed. Providing such numerical studies is of great importance in analyzing and optimizing of the bioreactor and can be beneficial for drug development industries by reducing time and design cost of the predesign process.

### Parametric Study

In this section, the result of changing different parameters affecting the efficiency of the perfusion bioreactor is investigated. Several parameters like perfusion rate, intra spheroid porosity, hydrogel porosity, spheroid diameter, saturated dissolved oxygen concentration, *V*_*m*_ and *K*_*m*_ in Michaelis-Menten kinetics are varied individually over the specified range while keeping other parameters at the fixed baseline values mentioned in Table 4. Porosity, considered as a microstructural property of the porous media, modifies the transport phenomena (Equation 3), effective diffusion coefficient (Equation 9) and permeability (Equations 12 and 13). Evaluation of *in vitro* hepatic spheroid porosity is quite challenging, and there is little information about that (Moussy, 2003; Khakpour et al., 2017). Additionally, the hepatic spheroid porosity is not a permanent value and varies as cells proliferate and rearrange during the culture period. Several methods have been developed for estimating the porosity of packed beds of monodispersed rigid spheres in which mean porosity values are associated with the processes of formation (Dullien, 2012). The range of porosities covers the range from, thinnest regular packing 0.48, very loose random packing 0.44, loose random packing 0.40–0.41, poured random packing 0.375–0.391 to minimum porosities in close random packing 0.36–0.37 and 0.26 in densest regular packing (hexagonal close pack) (Dullien, 2012). Porosity in hepatocytes spheroid can be lower than 0.26 since the cells are not rigid and can adhere together firmly (Khakpour et al., 2017). Hence, the presented parametric study is performed for three different spheroid porosity, ε_*sph*_, namely 0.2, 0.3, 0.5, which ε_*sph*_ = 0.2 is considered as the baseline value. Furthermore, since in larger spheroid diameter, cells especially in the core sections of the aggregates may suffer from severe oxygen limitation, oxygen concentration inside the different spheroid diameters is evaluated, and feasibility of reaching physiological oxygen concentration is analyzed. The parametric study was carried out for the last spheroid, which is considered as the severe case of study since its location is in the furthest distance from the inlet.

**Table 4:** Parameters, their respected default value and their range over which they were investigated in the parametric study.

**Parameter**	**Unit**	**Baseline value**	**Sweep range**
Saturated oxygen concentration, C_o2, sat_	μmol/L	280	100–300
Perfusion rate, Q	ml/min	1	0.1–2.70
Spheroid diameter, d_sph_	μm	200	100–400
Maximum consumption rate (Michaelis-Menten), V_m_	nmol/ (s cm^3^)	5	5–20
Concentration associated with V_m_/2 (Michaelis-Menten), K_m_	μmol/L	1	0.7, 1, and 7.8
Intra-spheroid porosity, ε_sph_		0.2	0.2–0.50
Hydrogel porosity, ε_hyd_		0.7	0.15–0.80

#### Dimensional Analysis

Dimensional analysis is carried out to investigate the dominance of parameters participating in the transport mechanism. The parameters are defined in Table 5, and their values are given in Table 6. The Peclet number compares the rate of convection to the diffusion for oxygen. In the present study, three Peclet numbers are defined: *Pe*_*sph*_, Peclet number for the intra spheroid section, *Pe*_*hyd*_, Peclet number for hydrogel part and *Pe*_*main*_, Peclet number for the main-stream section. The oxygen diffusion coefficient is acquired by Equation (9) based on the baseline porosity value (3 × 10^−10^ m^2^/s for ε = 0.2), which increases with an increase in porosity. Based on the low-velocity rate inside the spheroids and spheroid diameter of 200 μm (baseline values), the Peclet number is generally in a range from O(10^−2^) to O(10^−6^) obtained based on the reported range of perfusion rate and aggregate diameter (Table 4). Such low values of the Peclet number indicates that the transport mechanism in intra spheroid space is mainly diffusion dominant.

**Table 5:** Dimensional parameters used for evaluation of oxygen concentration distribution inside the bioreactor.

**Parameter**	**Definition**	**Description**
Péclet number (intraspheroid)	${}_{Pe_{sph}=\frac{u_{sph}D_{sph}}{D_{o,sph}}}$	$\frac{Convective\;transport\;rate}{Diffusive\;transport\;rate}$
Péclet number (hydrogel domain)	${}_{Pe_{hyd}=\frac{u_{hyd}L_{hyd}}{D_{o,hyd}}}$	$\frac{Convective\;transport\;rate}{Diffusive\;transport\;rate}$
Péclet number (main-stream domain)	${}_{Pe_{main}=\frac{u_{main}L_{main}}{D_{o,liq}}}$	$\frac{Convective\;transport\;rate}{Diffusive\;transport\;rate}$
Damköhler number	${}_{Da=\frac{V_{\max}D_{sph}^{2}}{D_{o,hyd}C_{o,sat}}}$	$\frac{Oxygen\;consumption\;rate}{External\;diffusion\;rate}$
Biomimicry index	_β_*Bm*__	$\frac{Spheroid\;volume\;with\;Co\;within\;in\;vivo\;range}{Total\;spheroid\;volume}$
Hypoxia index	_β_*Hpx*__	$\frac{Spheroid\;volume\;with\;Co\;<\;in\;vivo\;range}{Total\;spheroid\;volume}$

**Table 6:** Values obtained for dimensionless parameters considering baseline values in Table 4.

**Pe_sph_**	**Pe_hyd_**	**Pe_main_**	**Da**	**β_Bm_**	**β_Hpx_**
2e-3	1	500	4.0	0.69	0

The Peclet number for hydrogel section is computed mostly around O(1) by considering the aforementioned range for perfusion rate, element size 2 mm and the oxygen diffusion coefficient in hydrogel over 1.6 × 10^−9^ m^2^/s. The O(1) is denoting the comparable effects of diffusion and convection rate. Additionally, the Peclet number [O(100)] is also estimated for the main-stream section by considering 4 × 10^−3^ m as element size and 3.4 × 10^−9^ m^2^/s for the oxygen diffusion coefficient, showing the dominance of convection transport mechanism. Higher perfusion rate will decrease the oxygen concentration gradient inside the hydrogel section, which in turn results in the higher level of oxygen supply at the vicinity of the hepatic spheroids (Figure S2). The order of magnitude of Peclet numbers is O(10^−3^), O(1), and O(10^2^) for intra spheroid, hydrogel, and main-stream, respectively, representing the dominance of diffusion rate inside the spheroid and prevalence of the convection mechanism in the main-stream.

The relation between the oxygen reaction rate and diffusion rate is investigated through the Damkohler number, which compares the rate of oxygen diffusion rate from hydrogel to spheroid to the maximum reaction rate *V*_*m*_. By considering the aforesaid parameters, Da number varies from 3.2 to 5.5.

Besides, two more indexes are introduced here to show the conditions in which bioreactor provide a suitable microenvironment for the hepatocytes. Biomimicry index extracted from a similar method of previous studies carried out by Dulong and Legallais (2007) and Khakpour et al. (2017), indicating the ratio of regions in which hepatocytes are exposed to physiological level of oxygen supply (4–9%) (Khakpour et al., 2017), though providing a higher or lower level of oxygen supplies does not necessarily indicate total loss of hepatocyte functionalities (Tourlomousis and Chang, 2016a). Hypoxia index also illustrates the region in the spheroids where oxygen levels fall below 4% (42 μmol/L), which can be helpful in choosing appropriate design and working parameters of the bioreactor.

Next, the effect of changing various operational and design parameters affecting the efficiency of the bioreactor will be analyzed. Operational parameters like inlet oxygen concentration and perfusion rate can easily be modified to provide an optimized oxygen supply for the hepatocytes. Changing design parameters such as spheroid diameter, hydrogel porosity, and kinetic parameters is not straightforward since hepatocytes diameter is changing over culture time. Also, it is possible to choose different biocompatible hydrogel for encapsulating the cells, but hydrogel porosity ranges are not large (between 0.3 and 0.7) (Tan et al., 2013; Mai et al., 2014; Pacelli et al., 2018). Kinetic parameters like (*V*_*m*_, *K*_*m*_) are intrinsic to the cell type, and therefore, these parameters cannot be altered (Curcio et al., 2014).

**Figure 9:**
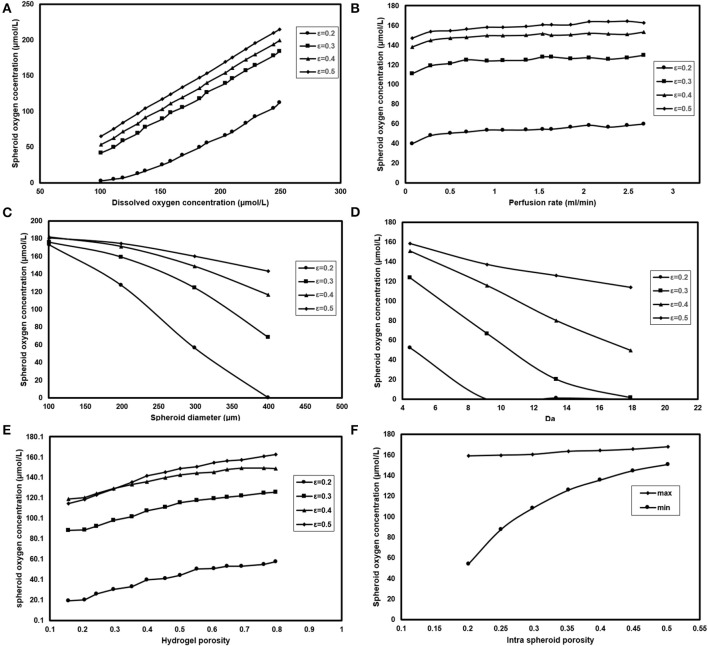
Analyzing the effect of different parameters on spheroid oxygen concentration. Measuring oxygen concentration level inside the spheroid for different **(A)** Dissolve oxygen concentration **(B)** Perfusion rate **(C)** Spheroid diameter **(D)** Da **(E)** Hydrogel porosity **(F)** Intra spheroid porosity.

Oxygen concentration at the inlet can be modified by modifying the oxygen saturation level in the perfusion media via changing the oxygen partial pressure of the incubator (Equation 14). Figure 9A displays the effect of changing oxygen partial pressure inside the incubator where intra spheroid oxygen level follows the similar increment of oxygen partial pressure, especially at higher porosities. Higher oxygen pressure will increase intra spheroid oxygen level. Hence in terms of dimensionless parameters, an increase of oxygen partial pressure from 100 to 300 μmol/L will result in elevated intra spheroid oxygen concentration with the almost similar linear trend. Additionally, supplying hepatocytes with higher oxygen level will push down the *Da*. A gradual increase in oxygen concentration from 100 to 300 μmol/L at ε_sph_ = 0.2 will cause a reduction in both *Da*, which falls from 8.3 to 3.3.

Perfusion rate, as the second operational parameters studied here, can be easily tuned by modifying the rotational speed of the peristaltic pump. Increasing the perfusion rate is accompanied by higher oxygen delivery to the cells (Figure 9B), but there should be a limit for the maximum value of the perfusion rate based on the shear stress threshold tolerated by hepatocytes. It can be noticed spheroid oxygen concentrations do not change significantly under the studied perfusion rate.

Spheroid diameter is changing, resulting from hepatocytes proliferation during the culture period. Hepatocytes in larger hepatic spheroids are more prone to lower oxygen levels considering the diffusion as the dominant oxygen transport mechanism. Figure 9C depicts the oxygen concentration level for spheroids of different sizes. Oxygen supply level in the smaller spheroids even at low porosities, based on the short diffusive penetration depth of oxygen, is almost in normoxic conditions. Increasing spheroid diameter from 100 to 400 μm will enhance β_*Hpx*_ from 0 to 0.64 showing that spheroids with >200 μm, especially at lower porosities are at the high risk of hypoxic conditions.

Measuring *K*_*m*_ value is quite challenging, which requires complex evaluation; hence, data reported for different values of *K*_*m*_ does not cover a large domain. Three values of 0.7, 1, and 7.8 μmol/L are chosen based on the values given by Hay (Dunn et al., 1991) and Khakpour et al. (2017). Figure 9D shows the dependency of hepatocytes oxygen concentration on *Da* variation for different aggregates porosities. Oxygen concentration level inside the spheroid is highly dependent on *V*_*m*_ values, where an increase from 5 to 20 nmol/(s-cm3) will contribute to around 30 % raise in β_*Hpx*_ values at a porosity of 0.2. The variation trend slows down at higher porosities. For instance, ε_*sph*_ = 0.5 is accompanied by 10% raise for β_*Hpx*_.

It is also possible to modify the porosity of the hydrogel by using different appropriate hydrogels or applying various conditions of hydrogel cross-linking (Tourlomousis and Chang, 2016b). Modifying the membrane porosity does not affect the perfusion rate of the medium, provided that the peristaltic pump circulates the media at the constant flow rate. However, a hydrogel with high porosity will reduce pressure drop requires for flow perfusion (Equation 13). Hydrogel with higher porosity provides less oxygen transport resistance, and thereby higher level of oxygen supply to the cells (Figure 9E) showing that changing hydrogel porosities will modify both maximum and minimum of oxygen concentration inside the hepatocytes. Increasing hydrogel porosity will cause a slight increment in biomimicry index (12%) at higher porosities and remain almost similar at ε = 0.2.

Effects of different spheroid porosities on the intra spheroid oxygen concentration are also investigated for two *K*_*m*_ values, i.e., 0.7, and 7.8 μmol/L representing the minimum, the maximum values of the oxygen concentration, respectively. Figure 9F represents the effects of changing intra spheroid porosity on the oxygen level inside the spheroid, where the oxygen level is much higher in aggregates with larger porosities, especially in minimum values. Higher spheroid porosity is correlated with a less number of cells in the aggregates with the specific diameter (Equation 8), which in turn leading to a less oxygen demand from the cells inside the spheroid. Furthermore, the effective diffusion coefficient increases with an increase in the porosity of the spheroids (Equations 9–11), resulting in less mass transfer (oxygen delivery) resistance to the cells. Increasing ε_*sph*_ from 0.2 to 0.5 resulted in increasing of *Pe*_*sph*_ level (from 2 × 10^−3^ to 7 × 10^−2^) and decreasing of β_*Bm*_ (from 0.13 to 0).

## Conclusion

Here in the presented study, a 3D computational model of perfusion bioreactor containing hepatocyte spheroids encapsulated in the hydrogel was investigated, and the influence of different parameters affecting the intra spheroid oxygen concentration was inspected. A full hydrodynamic model of medium flow inside the bioreactor confirmed that shear stress imposed on hepatocytes was far below the detrimental threshold (0.03 Pa). Hosting spheroids inside the hydrogel would protect cells from shear stress, and hence, in conditions in which higher oxygen level would be required, such as aggregates with the larger diameters or the higher number of cells, the higher level of flow rates could be applied.

Additionally, the presented model incorporated mass transfer of various metabolites (i.e., oxygen, albumin, glucose, glutamine, ammonia, and urea) known as crucial parameters in liver specific-functions. The distribution of the oxygen concentration inside the bioreactor was obtained. Albumin, glucose, glutamine, ammonia, and urea distribution inside the bioreactor were also evaluated. Predicted results of the albumin production represented a good agreement with the experimental data. Results of glucose and glutamine predictions would be of great importance in generating insight for researchers by providing a baseline index prior to any fabrication. For example, obtained results indicated the low glucose consumption rate of the hepatocytes, would be beneficial in estimating the minimum necessary glucose requirement of the hepatocytes. Ammonia is a toxic compound which is eliminated by hepatocytes from the circulating system through the urea cycle. Ammonia in the presented model had two terms of elimination and generation. It was removed by converting to urea and generated in the glutamine synthesis. Ammonia to urea conversion was modeled through four enzymatic reactions. The detailed concentration of each participating metabolite was obtained by solving 12 coupled differential equations. Estimated results obtained from urea synthesis could be further applied to predict the diseases relating to the urea cycle, such as inborn urea cycle disorders.

Since oxygen tension is a critical parameter in viability and maintaining the liver-specific functionalities of the cells, in the next step of this study, a parametric study was carried out based on the oxygen concentration and the effect of different design conditions on the oxygen level inside the hepatic spheroids was investigated. Oxygen tension inside the hepatic aggregates were obtained for the different perfusion rates. Since the dominant transport mechanism in the main-stream is convection [*Pe*_*main*_ O(100)], higher perfusion rates would provide higher oxygen supply to the vicinity of the spheroids. Among different studied design parameters, saturation oxygen concentration at the inlet was the most influential parameter which could be tuned based on the required level of the oxygen in the system.

Numerical results showed that spheroids with smaller diameters were less prone to hypoxia conditions based on their short diffusive penetration depth and for the spheroids with the diameter larger than 200 μm, the precise control of oxygen delivery must be applied. Transport mechanism inside the spheroids was diffusion dominant [*Pe*_*sph*_ O(10^−3^)]. Maximum oxygen consumption rate, *V*_*m*_, also played an important role in determining the oxygen distribution inside the spheroids. High *V*_*m*_ values could lead to anoxia condition, which was accompanied by a high level of *Da*. Spheroid porosity was shown to be an essential factor affecting both the number of the hepatocytes and the oxygen concentration distribution inside the spheroid. It is worth mentioning that hydrogel porosity noticeably affected the intra spheroid oxygen concentration. Both convection and diffusion had a comparable dominance in the hydrogel section [*Pe*_*hyd*_ O(1)]; hence, mass transfer resistance caused by hydrogel porosity was found to be a pivotal parameter affecting the intra spheroid oxygen concentration. This multi-scale computational system allows the study of some main liver-specific functions and optimization of bioreactor characteristics based on efficient oxygen transport. We also envision to develop a more complex model of metabolisms and include the other liver-specific metabolisms to the presented model.

## Data Availability

The raw data supporting the conclusions of this manuscript will be made available by the authors, without undue reservation, to any qualified researcher.

## Author Contributions

FS and BF conceived of the presented idea. FS developed the theory and performed the computations. She developed the theoretical formalism, performed the analytic calculations, and performed the numerical simulations. BF and KF verified the analytical methods and supervised the findings of this work. FS wrote the manuscript under the supervision of BF and KF. All authors discussed the results and contributed to the final manuscript.

### Conflict of Interest Statement

The authors declare that the research was conducted in the absence of any commercial or financial relationships that could be construed as a potential conflict of interest.
