# Supplementary material for: Numerical Investigations of Hepatic Spheroids Metabolic Reactions in a Perfusion Bioreactor
Source: Front Bioeng Biotechnol. 2019 Sep 12;7:221. doi: 10.3389/fbioe.2019.00221 (PMC6751279; doi:10.3389/fbioe.2019.00221)
Supplement: Supplementary file 1 [file Table_1.docx]

# Supplementary Material


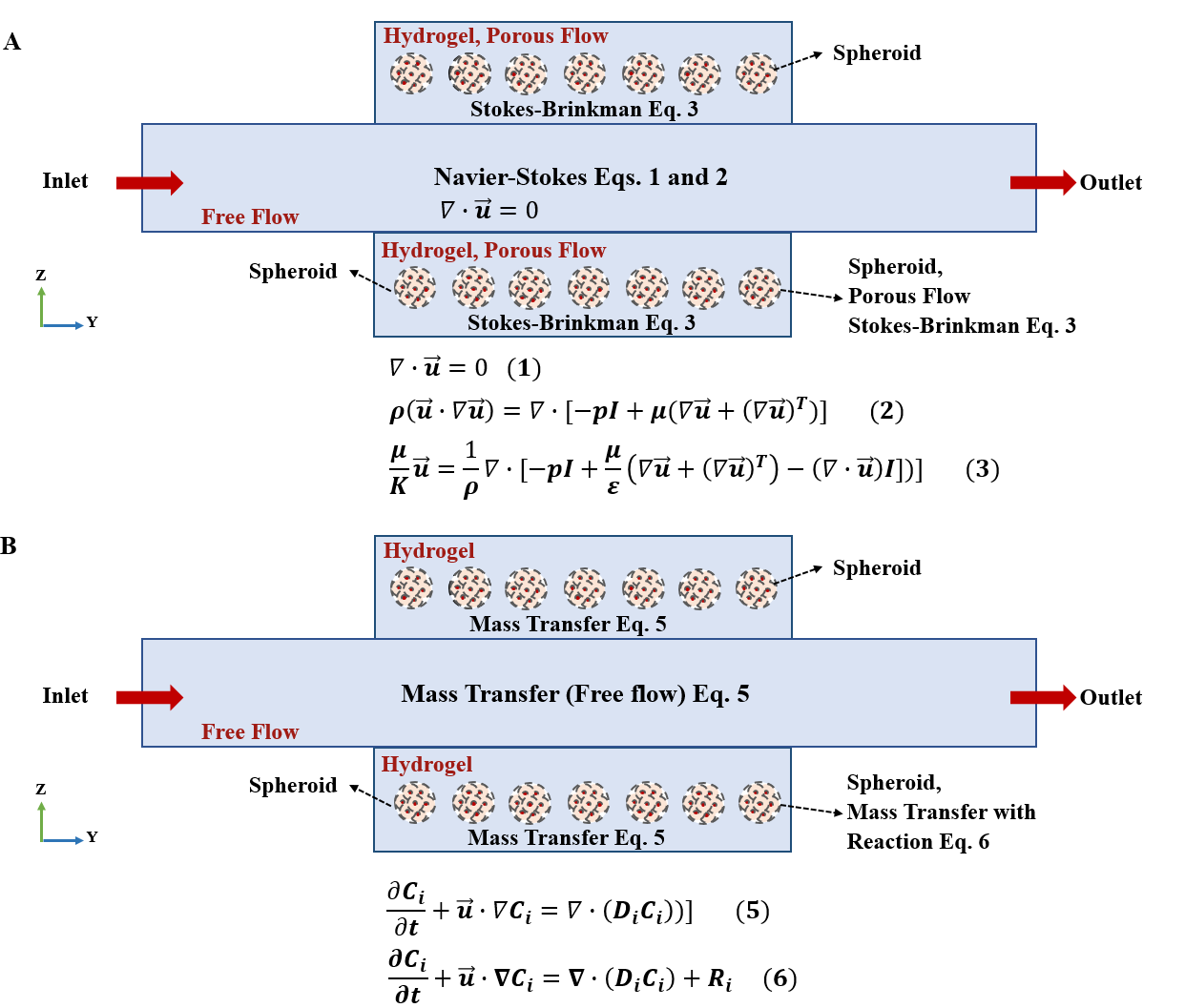


**Supplementary Figure S1**. Schematic of the different domains along with the respected governing equations used for the simulation. The bioreactor consists of three main compartments, i.e., two hydrogel sections and main-stream channel. Schematic front view of the bioreactor along with governing equations for **(A)** hydrodynamic and **(B)** Mass transfer modeling.


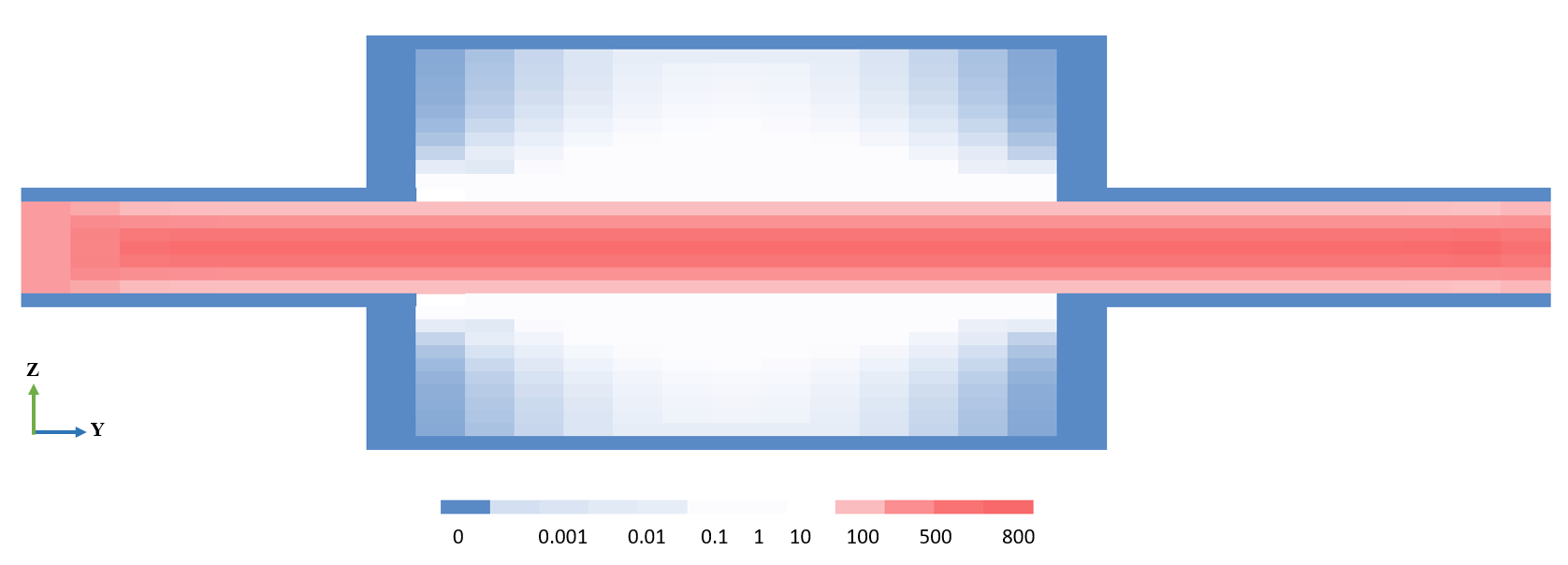


**Supplementary Figure S2**. Heat map plot of Peclet number distribution at *x=0* surface, comparing the rate of convection to the diffusion for oxygen. The order of magnitude of Peclet numbers is O(10^-3^), O(1) and O(10^2^) for intra spheroid, hydrogel, and main-stream, respectively, representing the dominance of diffusion rate inside the spheroid and prevalence of the convection mechanism in the main-stream.

## Urea production

Urea synthesis is a cycle of biochemical reactions happens mainly in the liver in which highly toxic ammonia converts to urea through a urea cycle pathway [10]. The urea cycle pathway based on its main enzyme reactions, is generally divided into four stages: Ornithine transcarbamoylase, Argininosuccinatesynthetase, Argininosuccinate and Arginase [35] (Fig. 4). Ammonia ions are converted to carbamoyl phosphate through a reaction called carbamoyl phosphate synthesis before entering the hepatocytes. In the first enzymatic reaction inside the hepatocyte, the carbamoyl phosphate is converted to citrulline and one phosphate group is released. Then, citrulline accompanied by aspartate is catalyzed by argininosuccinate synthetase and donated to argininosuccinate which in turn is led to the formation of fumarate and arginine by argininosuccinase. Argininge undergoes cleavage by arginase to form urea and ornithine. Generated ornithine will again begin to participate in the urea cycle and urea will excrete out of the hepatocyte [15].

The rate equation for the ornithine transcarbamoylase, argininosuccinatesynthetase, argininosuccinate and are given in the following equations, respectively:

| $v_{1}=\frac{\left\{k_{1}k_{2}k_{3}k_{4}\left[CP\right]\left[O\right]-k_{2}k_{4}k_{6}k_{8}\left[P\right]\left[C\right]\right\}\left[E_{1}\right]_{0}}{\begin{matrix} \left\{k_{2}k_{7}\left(k_{4}+k_{5}\right)+k_{1}k_{7}\left(k_{4}+k_{5}\right)\left[CP\right]+k_{2}k_{8}\left(k_{4}+k_{5}\right)\left[P\right]+\right. \\ k_{3}k_{5}k_{7}\left[O\right]+k_{2}k_{4}k_{6}\left[C\right]+k_{1}k_{3}\left(k_{5}+k_{7}\right)\left[CP\right]\left[O\right]+ \\ k_{6}k_{8}\left(k_{2}+k_{4}\right)\left[P\right]\left[C\right]+k_{1}k_{4}k_{6}\left[CP\right]\left[C\right]+k_{1}k_{3}k_{6}\left[CP\right]\left[O\right]\left[C\right]+ \\ k_{3}k_{5}k_{8}\left[O\right]\left[P\right]+\left.k_{3}k_{6}k_{8}\left[O\right]\left[P\right]\left[C\right]\right\} \end{matrix}}$ | | (A-1) |
| --- | --- | --- |
|  | (A-2) | |
| $v_{3}=\frac{\left\{k_{1}k_{3}k_{5}\left[AS\right]-k_{2}k_{4}k_{6}\left[F\right]\left[A\right]\right\}\left[E_{3}\right]_{0}}{\begin{matrix} \left\{k_{5}\left(k_{2}+k_{3}\right)+k_{1}\left(k_{3}+k_{5}\right)\left[AS\right]+k_{2}k_{4}\left[F\right]+k_{6}\left(k_{2}+k_{3}\right)\left[A\right]+\right. \\ k_{4}k_{6}\left[A\right]\left[F\right]+\left.k_{1}k_{4}\left[AS\right]\left[F\right]\right\} \end{matrix}}$ | (A-3) | |
| $v_{4}=\frac{k_{1}k_{3}k_{4}\left[A\right]\left[E_{4}\right]_{0}}{k_{4}\left(k_{2}+k_{3}\right)+k_{5}\left(k_{2}+k_{3}\right)\left[O\right]+k_{1}\left(k_{3}+k_{4}\right)\left[A\right]}$ | (A-4) | |

which for each rate equation, constants and enzymatic activity should be identified. Reaction constants and enzymatic activity of each relation are given in Supplementary Table 2. and Supplementary Table 2., respectively.

| **Supplementary Table 3.** Constants of each urea reaction rate equations [35]. | | | | | |
| --- | --- | --- | --- | --- | --- |
| Reaction | Constant  [SI unit] | Value | Reaction | Constant  [SI unit] | Value |
| Ornithine Transcarbamoylase | k_1_ [M^-1^S^-1^] | 1.7×10^7^ | Argininosuccinate synthetase | k_9_ [S^-1^] | 5.0×10 |
|  | k_2_ [S^-1^] | 6.3×10 |  | k_10_ [M^-1^S^-1^] | 6.4×10^5^ |
|  | k_3_ [M^-1^S^-1^] | 2.1 ×10^6^ |  | k_11_ [S^-1^] | 5.0×10 |
|  | k_4_ [S^-1^] | 1.0×10^3^ |  | k_12_ [M^-1^S^-1^] | 1.7 ×10^5^ |
|  | k_5_ [S^-1^] | 3.0×10^3^ | Argininosuccinase | k_1_ [M^-1^S^-1^] | 2.7 ×10^6^ |
|  | k_6_ [M^-1^S^-1^] | 9.0×10^4^ |  | k_2_ [S^-1^] | 7.0×10 |
|  | k_7_ [S^-1^] | 2.6×10^3^ |  | k_3_ [S^-1^] | 7.5 ×10 |
|  | k_8_ [M^-1^S^-1^] | 5.0×10^5^ |  | k_4_ [M^-1^S^-1^] | 1.5×10^6^ |
| Argininosuccinate synthetase | k_1_ [M^-1^S^-1^] | 2.4 ×10^5^ |  | k_5_ [S^-1^] | 1.1×10^3^ |
|  | k_2_ [S^-1^] | 2.3×10 |  | k_6_ [M^-1^S^-1^] | 7.0×10^5^ |
|  | k_3_ [M^-1^S^-1^] | 3.5 ×10^4^ | Arginase | k_1_ [M^-1^S^-1^] | 1.0 ×10^7^ |
|  | k_4_ [S^-1^] | 1.0×10 |  | k_2_ [S^-1^] | 5.4×10^4^ |
|  | k_5_ [M^-1^S^-1^] | 4.8×10^5^ |  | k_3_ [S^-1^] | 5.3 ×10^3^ |
|  | k_6_ [S^-1^] | 1.0×10 |  | k_4_ [S^-1^] | 3.0×10^4^ |
|  | k_7_ [S^-1^] | 2.0×10 |  | k_5_ [M^-1^S^-1^] | 1.0×10^7^ |
|  | k_8_ [M^-1^S^-1^] | 8.9×10^5^ |  |  |  |

| **Supplementary Table 2.** Enzymes activity participating in the urea cycle [35]. | | | |
| --- | --- | --- | --- |
| Enzyme | Total activity (I. U./ kg liver) | Total enzyme (mg/ kg liver) | Concentration (mol/lit) |
| Ornithine Transcarbamoylase | 1.1×10^5^ | 1.4×10^2^ | 2.6×10^-6^ |
| Argininosuccinatesynthetase | 1.4×10^3^ | 3.5×10^2^ | 4×10^-6^ |
| Argininosuccinase | 5×10^3^ | 2.2×10^2^ | 2.2×10^-6^ |
| Arginase | 9.2×10^5^ | 5.1×10^2^ | 8.9×10^-6^ |

From Eqs. A1-A4 concentration of the metabolites participating in the urea cycle can be obtained via the solving the following coupled differential equations simultaneously [2]:

| $\frac{d\left[C\right]}{dt}=v_{1}-v_{2}$ | (A-5) |
| --- | --- |
| $\frac{d\left[AS\right]}{dt}=v_{2}-v_{3}$ | (A-6) |
| $\frac{d\left[A\right]}{dt}=v_{3}-v_{4}$ | (A-7) |
| $\frac{d\left[O\right]}{dt}=v_{4}-v_{1}$ | (A-8) |
| $\frac{d\left[U\right]}{dt}=+v_{4}-k_{U_{tp}}\times U_{tp}$ | (A-9) |
| $\frac{d\left[CP\right]}{dt}=k_{NH_{4}}NH_{4-c}^{+}-v_{1}$ | (A-10) |
| $\frac{d\left[ATP\right]}{dt}=k_{ATP}^{*}\left[Y\right]_{pool}-v_{2}$ | (A-11) |
| $\frac{d\left[ASP\right]}{dt}=k_{ASP}^{*}\left[Y\right]_{pool}-v_{2}$ | (A-12) |
| $\frac{d\left[PP\right]}{dt}=-k_{PP}^{*}\left[PP\right]+v_{2}$ | (A-13) |
| $\frac{d\left[AMP\right]}{dt}=-k_{AMP}^{*}\left[AMP\right]+v_{2}$ | (A-14) |
| $\frac{d\left[F\right]}{dt}=-k_{F}^{*}\left[F\right]+v_{3}$ | (A-15) |
| $\frac{d\left[P\right]}{dt}=-k_{P}^{*}\left[P\right]+v_{1}$ | (A-16) |

The initial value of each metabolite is given in Supplementary Table 3.

| **Supplementary Table 3.** Initial concentration of metabolites used in this study [19]. | | |
| --- | --- | --- |
| Abbreviation | Full name | Initial concentration (M) |
| C | Citrulline | 1× 10^-7^ |
| AS | Argininosuccinate | 1× 10^-5^ |
| A | Arginin | 1× 10^-7^ |
| O | Ornithine | 4.5× 10^-4^ |
| U | Urea | 1× 10^-5^ |
| CP | Carbamoyl phosphate | 1× 10^-4^ |
| ATP | Adenosine triphosphate | 1× 10^-3^ |
| ASP | Aspartate | 1× 10^-3^ |
| PP | Pyrophosphate | 1× 10^-5^ |
| AMP | Adenosine monophosphate | 1× 10^-5^ |
| F | Fumarate | 1× 10^-5^ |
| P | Phosphate | 1× 10^-5^ |

## Reynolds number calculation

The perfusion rate is swept from 0.1-2.7 ml/min, therefore,

$$\boldsymbol{Q}=\mathbf{0}.\mathbf{1}\frac{\boldsymbol{ml}}{\boldsymbol{min}}\Rightarrow\boldsymbol{Re}=\frac{\boldsymbol{\rho}\vec{\boldsymbol{u}}\boldsymbol{L}}{\boldsymbol{\mu}}=\frac{\mathbf{1000}\times\mathbf{4}\times\mathbf{10}^{-\mathbf{4}}\times\mathbf{2}\times\mathbf{10}^{-\mathbf{3}}}{\mathbf{1}.\mathbf{002}\times\mathbf{10}^{-\mathbf{3}}}=\mathbf{0}.\mathbf{833}$$

$$\boldsymbol{Q}=\mathbf{1}\frac{\boldsymbol{ml}}{\boldsymbol{min}}\Rightarrow\boldsymbol{Re}=\frac{\boldsymbol{\rho}\vec{\boldsymbol{u}}\boldsymbol{L}}{\boldsymbol{\mu}}=\frac{\mathbf{1000}\times\mathbf{4}\times\mathbf{10}^{-\mathbf{3}}\times\mathbf{2}\times\mathbf{10}^{-\mathbf{3}}}{\mathbf{1}.\mathbf{002}\times\mathbf{10}^{-\mathbf{3}}}=\mathbf{8}.\mathbf{33}$$

$$\boldsymbol{Q}=\mathbf{2}.\mathbf{7}\frac{\boldsymbol{ml}}{\boldsymbol{min}}\Rightarrow\boldsymbol{Re}=\frac{\boldsymbol{\rho}\vec{\boldsymbol{u}}\boldsymbol{L}}{\boldsymbol{\mu}}=\frac{\mathbf{1000}\times\mathbf{0.011}\times\mathbf{2}\times\mathbf{10}^{-\mathbf{3}}}{\mathbf{1}.\mathbf{002}\times\mathbf{10}^{-\mathbf{3}}}=\mathbf{22}.\mathbf{45}$$

## Specious continuity and stress continuity boundary conditions

As shown in the Fig. 3B, red dash line indicates the boundaries between the hydrogel and the free flow and hydrogel and spheroids. Specious continuity and stress continuity boundary conditions were applied on these boundaries, i.e.

for specious, at the boundary

$$C_{i.shperoid}=C_{i.hydrogel}$$

and for stress continuity

$$\boldsymbol{n}\cdot(\boldsymbol{T}_{\mathbf{2}}-\boldsymbol{T}_{\mathbf{1}})=0$$

Where **T** denotes stress tensors. This boundary condition applies to interfaces shown by red dash line. Therefore, for interface between main flow and hydrogel, indexes 1 and 2 apply to main flow and hydrogel sections, respectively. For the interface between hydrogel and spheroids, 1 denotes hydrogel and 2 indicates spheroid parts.

$$-\boldsymbol{\nabla}\boldsymbol{p}-\frac{\boldsymbol{\mu}}{\boldsymbol{\kappa}}\vec{\boldsymbol{u}}+\frac{\boldsymbol{\mu}}{\boldsymbol{\varepsilon}}\boldsymbol{\nabla}^{\mathbf{2}}\vec{\boldsymbol{u}}=\mathbf{0}$$
